# Statistical Analysis of Morphological Characteristics of Inconel 718 Formed by High Deposition Rate and High Laser Power Laser Cladding

**DOI:** 10.3390/ma17030638

**Published:** 2024-01-28

**Authors:** Yanhua Bian, Xiuli He, Chongxin Tian, Jianhao Guo, Bo Chen, Binxin Dong, Shaoxia Li, Gang Yu

**Affiliations:** 1 Institute of Mechanics, Chinese Academy of Sciences, Beijing 100190, China; 2 Center of Materials Science and Optoelectronics Engineering, University of Chinese Academy of Sciences, Beijing 100049, China; 3 School of Engineering Science, University of Chinese Academy of Sciences, Beijing 100049, China; 4 China Railway 14th Bureau Groupment Shield Construction Engineering Co., Ltd., Nanjing 210000, China; 5 Guangdong Aerospace Research Academy, Guangzhou 511458, China

**Keywords:** laser cladding, Inconel 718, clad morphology, linear regression analysis, high deposition rate

## Abstract

Laser cladding is one of the emerging additive manufacturing technologies and has been adopted in various industrial fields. In this study, the morphological characteristics of a single clad of Inconel 718 manufactured by coaxial laser cladding with high laser power from 4200 W to 5400 W, powder feeding rate from 25 g/min to 50 g/min, and cladding speed from 20 mm/s to 50 mm/s are studied. The cross-section of the melt pool is analyzed and classified by type into three types: shallow dilution, flat dilution, and fluctuating dilution. Nine parameters are designed to describe the morphological characteristics of the clad, and the corresponding linear regression models are developed to establish a quantitative relationship between the combined process parameters and morphological characteristics. The results indicate that the total area of the cross-section *A*, the clad area above the substrate *A_c_*, the area of the molten substrate *A_m_*, the total height of the cross-section *H*, the height of the clad above the substrate *h_c_*, the penetration depth *h_m_*, the clad width *W*, the dilution ratio *D*, and the wetting angle *θ* are determined by complex coupling of energy input and mass accumulation, and they are proportional to *PF*^0.4^/*V*, *P*^0.5^*F*/*V*, *P*/*F*^0.2^/*V*^0.4^, *P*^2^*F*^0.6^/*V*, *PF*^0.7^/*V*, *P*^2^/*F*/*V*^0.3^, *P*/*V*^0.8^, *P*/*FV*^0.2^, and *PF*^7^/*V*^0.8^, respectively. The large linear regression coefficients and the analysis residuals indicate the high reliability of the statistical linear regression models. This work aims to provide a comprehensive understanding of the influence of the main processing parameters on the morphological characteristics of the clad, which is of great value in providing a reference and laying a basis for the practical application of laser cladding technology at a high deposition rate.

## 1. Introduction

Inconel 718 (In718), with excellent mechanical properties of strength, creep resistance, and good fatigue life at high temperatures up to 700 °C, is a nickel-based super alloy of great importance in the aerospace and nuclear industries and automotive applications [1,2]. Laser cladding with coaxial powder feeding is one of the most widely used additive manufacturing (AM) technologies and can be used to manufacture complex components and large-scale parts without other specialized tooling. As a consequence, the application potential of In718 is further explored with the use of laser cladding technology.

In the laser cladding process, the single track is the basic unit of the building components. The morphological characteristics of the single track, i.e., width, height, depth, heat affected zone (HAZ), and wetting angle, are the basic properties in the layer-by-layer manufacturing process, which can be used for determining the process parameters of multi-track and multi-layer overlapping. In addition, morphological characteristics can be employed to measure the bonding strength of the coating adhering to the substrate, estimate the crack behavior of the clad [3], and evaluate the growth direction and growth mode of the microstructure [4]. Therefore, it is of great significance to conduct a sufficient study on the morphology of the single cladding track.

The cladding process is accomplished by the complicated and mutual coupling physical phenomena of laser energy input and powder mass addition, including the laser–powder interplay in the transport process, heat and mass transfer, thermo-capillary convection, and rapid solidification in the melt pool. Consequently, there are many parameters, such as laser power, beam spot diameter, absorption coefficient, cladding speed, and powder feeding rate, that can give rise to enormous differences in the morphological characteristics of the clad. Therefore, the establishment of quantitative relationships between multiple process parameters and the morphological characteristics of the clad is the primary problem to be settled in the study of cladding technology and has attracted numerous attention to this research. In summary, there are two main approaches in the literature aimed at solving this issue. The first one refers to physical process models, in which extremely complicated physical phenomena, including the heat transfer among the laser beam, the substrate, and the powder and mass transfer between the powder flow and the melt pool surface, are considered, aiming at explaining the dynamic evolution mechanism of the gas–liquid interface and solid–liquid interface and the induced clad morphology [5,6,7,8]. Although physical models [5,6,7,8] along with numerical calculations are effective in describing the influence of processing parameters on the clad morphology, the calculation of these models requires a lot of time and resources, and the complexity of these models makes this method unfriendly to engineering applications.

The other valid method is to establish a phenomenological model, accompanied by an analysis method of empirical statistics, which is based on a great number of experiments. Subsequently, a process map of the morphological characteristics of the clad can be created. Zhao et al. [9] employed the method of analysis of variance (ANOVA) to investigate the effect of process parameters on the clad geometry with the help of variance analysis based on 125 groups of single-track cladding experiments with laser power ranging from 350~450 W, powder feeding rate of 8.26 ~13.07 g/min, and cladding speed of 5~7 mm/s. In reference [10], the effect of three essential process parameters on the geometric characteristics of cold metal transfer welding of the austenitic stainless-steel material 316 L substrate have been studied with the use of a genetic algorithm. Pant et al. [11] employed the artificial neural network method to study the height and width of the cladding layer and the powder capture efficiency. Hao et al. [12] employed support vector regression (SVR) and back-propagation neural network, optimized by particle swarm optimization (PSO-BPNN), and extreme gradient boosting (XGBoost) methods, to build the prediction model between the bead morphology and process parameters, and the credibility of the models was confirmed by R2 values. Costa et al. [13] developed a linear regression method between morphological characteristics and the complex process parameter related to the main processing parameters of laser power, scanning speed, and powder feeding rate {*P*, *S*, *F*}. The complex parameter was written as *P*^α^*F*^β^*V*^γ^, in which the indices α, β, and γ can be obtained by the trial-and-error method. The form of the combined parameters and index of the processing parameters can be used to analyze the formation mechanism of the morphological characteristics of the clad. With the convenience of establishing the quantitative relationship between process parameters and the morphological characteristics of the clad, this linear regression method has been widely cited by the literature [14,15,16,17,18,19,20,21,22,23,24,25]. Ilanlou et al. [14] employed the linear regression and genetic optimization to establish the quantitative relationship between compound variable *P*^α^*F*^β^*V*^γ^ and geometric characteristics, in which *P*^α^*F*^β^*V*^γ^ is participating in Y = ax + b. Bax et al. [15] presented an unambiguous measure to quantify the shape of single tracks and provided a general tool to generate process parameter maps. Indeed, the results showed that the clad height is linearly proportional to *F*/*V* and the width to *P*/*V*^1/2^.

In summary, there are three main problems to be addressed in empirical-statistical models. First, the clad geometry is represented by a combination of various parameters, and the formation mechanism is different for each feature represented by each parameter. However, a full description of the clad geometry in the literature is insufficient. Second, most of these conventional laser cladding methods for IN718 have relatively low deposition rates, normally lower than 0.5 kg/h [26], which makes the processing efficiency of laser cladding technology much lower compared to other technology [27]. Third, due to the limitations of experimental conditions or laser technology, the laser power in previous studies was typically lower than 2500 W, and the cladding speed is below 20mm/s. As a result, the developed models in previous literature are unable to evaluate and predict the geometric morphology of the clad over a larger process range.

In order to develop a linear regression model within a high deposition rate and high-power laser cladding, a theoretical and experimental study of the coaxial laser cladding process with a high-powder feeding rate ranging from 25 g/min to 50 g/min, high laser power input ranging from 4200 W to 5400 W, and cladding speed from 20 mm/s to 50 mm/s is presented. First, employing a high-power laser with the maximum output power of 10 kW, process experiments with high-throughput process parameters input of laser power, powder feeding rate, and cladding speed are performed. Second, nine parameters are employed to describe the morphological characteristics of the clad. The quantitative analysis of the relationship between the processing parameters and complete morphological characteristics of the clad is carried out by establishing linear regression models, which are accompanied by the correlation and residual analysis. Finally, the formation mechanism of geometrical characteristics of the clad is fully discussed. This paper aims to provide an easy way for the quantitative guidance on geometric morphology for laser cladding to promote efficiency and accommodate practical engineering applications.

## 2. Experimental Setup

**Figure 1:**
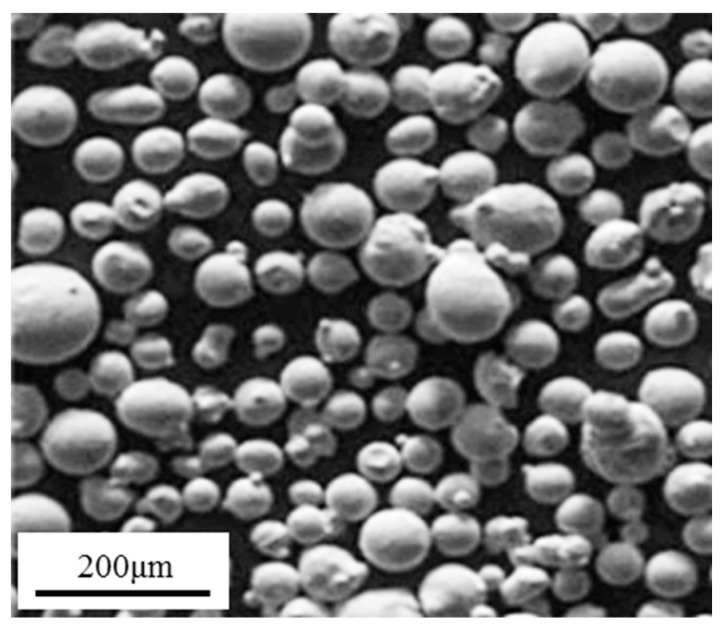
SEM morphology of the In718 powder.

In this study, the gas atomized powder of In718 with particles sized 48 μm to 143 μm was used to conduct the single-track cladding experiments, and its morphology is shown in Figure 1. Table 1 shows the chemical composition of In718. In718 alloy of 100 mm × 40 mm × 40 mm in dimension with the same chemical composition as the powder was employed as the substrate. Before the experiment, the substrate was cleaned with ethanol and then ground using a series of silicon carbide (SiC) paper of different grades (40–400#), and the powder was dried in a drying oven at 140 °C for 60 min and cooled at room temperature.

**Figure 2:**
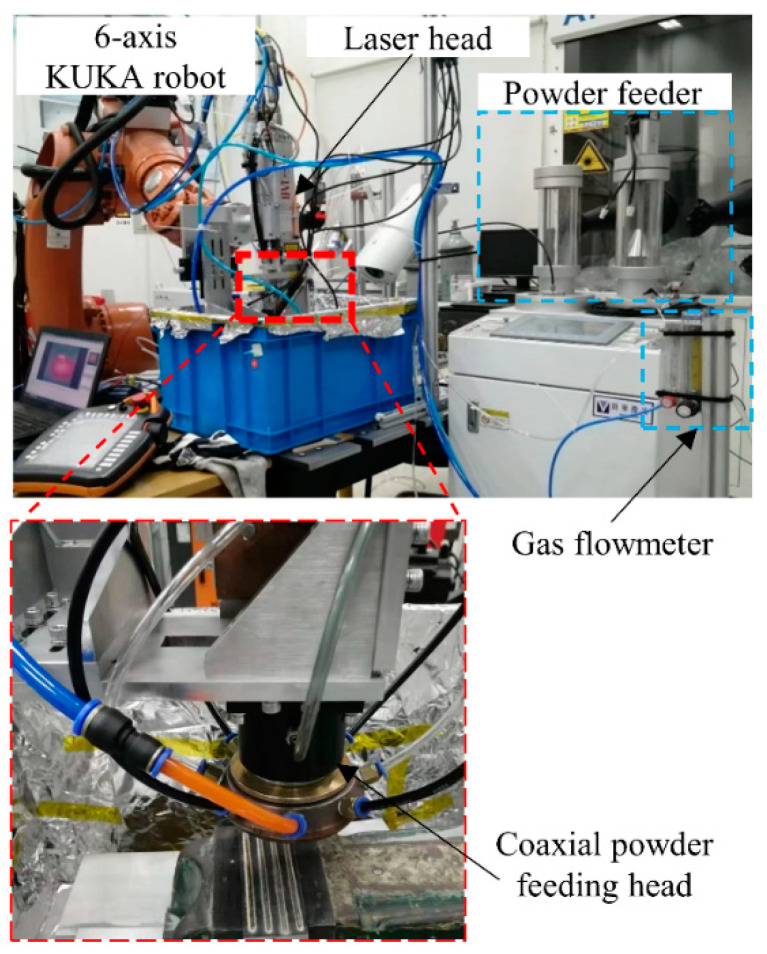
Experimental setup of laser cladding.

Single-track cladding experiments were carried out using an IPG fiber laser system with a maximum output power of 10 kW and a wavelength of 1070 nm. The laser spot diameter was set as 4 mm with a defocus distance of 40mm from the substrate. A coaxial powder feeding nozzle was used as the powder and atmosphere gas feeder, and argon gas was employed as a shielding and powder-carrying gas. The actual image of the experimental set was shown in Figure 2, and the detailed structure of the nozzle can be obtained from our previously published paper [28]. Both the laser and the nozzle were fixed to a six-axis KUKA robot system. The processing conditions were listed as follows: the laser power (P) was 4200–5400 W with an interval of 300 W; the laser scanning speed (V) was 20–50 mm/s with an interval of 10 mm/s; the powder feeding rates (F) were 25 g/min, 35 g/min, and 50 g/min; and the inner shielding gas flow rate, outer shielding gas flow rate, and carrying gas flow rate were 15 L/min, 15 L/min, and 8 L/min, respectively.

After laser cladding, all the samples were ultrasonically cleaned with 95% ethanol for 30 min. A Dino-Lite digital microscope AM4115ZT (Dino-Lite, Taiwan, China)with a magnification range from 20 × to 220 × was used to capture the surface morphology of the clad. Cross-sectional morphologies of the clad were obtained by cutting the samples from the longitudinal direction of the cladding, and they were then subjected to grinding using a series of silicon carbide (SiC) paper of different grades (240–2000#) and polished using diamond suspensions for the metallographic sample preparation. The polished samples were corroded using an etching solution of 5 g CuCl_2_, 100 mL C_2_H_5_OH, and 100 mL HCl for 60 s and subsequently washed with 99% ethanol. The cross-sectional morphological characteristics of the clads were obtained by using a Dino-Lite AM4115ZT optical microscope.

**Figure 3:**
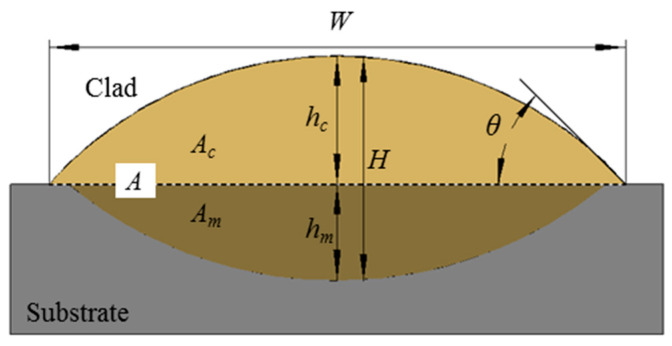
Schematic diagram of the geometrical characteristic of the clad: *W*: the clad width, *A_c_*: the area of the clad above the substrate, *A_m_*: the area of the molten in the substrate, *A*: the total area of the clad, *H*: the total height of the clad, *h_c_*: the clad height above the substrate, *h_m_*: the penetration depth, *θ*: the wetting angle, *θ =* arctan(*2h_c_*/*W*) [25], *D*: the dilution ratio *D = h_c_*/*H* [21].

Nine geometrical parameters (the clad width (*W*), the area of the clad above the substrate (*A_c_*), the area of the molten substrate (*A_m_*), the total area of the cross-section (*A*), the total height of the cross-section (*H*), the clad height above the substrate (*h_c_*), the penetration depth (*h_m_*), the clad angle (*θ*), and the dilution ratio (*D*)) of the cross-section morphology of the clad illustrated in Figure 3 were measured using imageJ2 software.

## 3. Results

### 3.1. Morphology of the Clad

**Figure 4:**
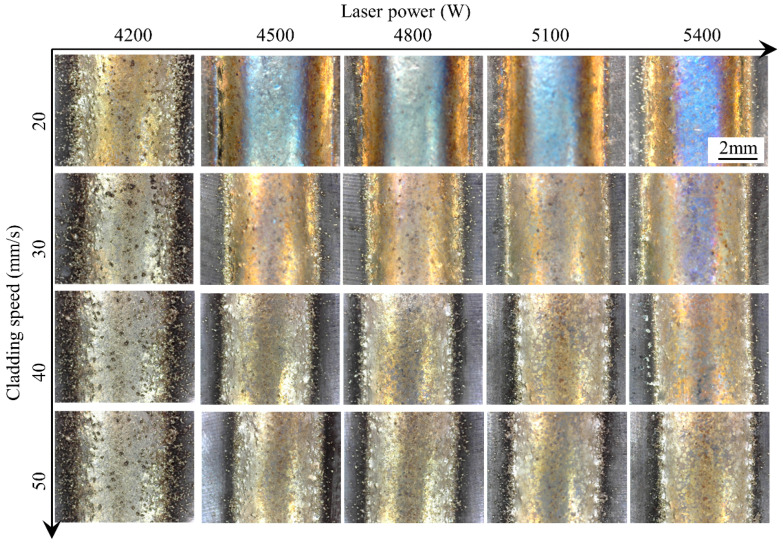
Top surface morphologies of the clads under different laser powers and cladding speeds.

**Figure 5:**
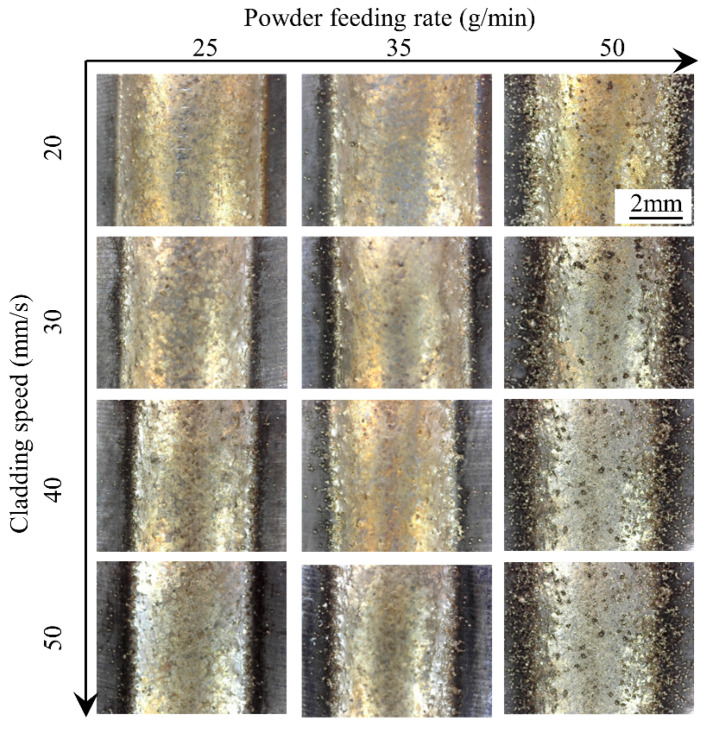
Top surface morphologies of the clads under different powder feeding rates and cladding speeds.

Figure 4 shows the single cladding tracks conducted under a powder feeding rate of 50 g/min, and Figure 5 shows the single cladding tracks conducted under laser power of 4200 W. The global features and surface states of the clad are evaluated first. Judging from the top view of the clad, stable cladding can be achieved. The unmelted powder can be observed and becomes the main index for evaluating the quality of the process. The number of unmelted particles adhered to the surface of the clad decreases with the increase in laser power and increases with the increase in cladding speed. When the powder feeding rate is 50 g/min and the laser power is 4200 W, a lot of unmelted particles are bonded to the surface of the clad and its surrounding areas, which indicates that the laser energy is insufficient relative to a high powder feeding rate. In the cladding process, the powder is heated from two aspects: absorption of laser energy during the transmission process between the substrate and the molten pool and energy absorption after entering the melt pool. Increasing the laser power, on the one hand, increases the energy absorbed by the powder during the flight process and, on the other hand, increases the temperature of the molten pool. As a result, increasing the laser power is an important means to obtain a smooth cladding surface morphology.

### 3.2. Geometrical Characteristics of the Clad

**Figure 6:**
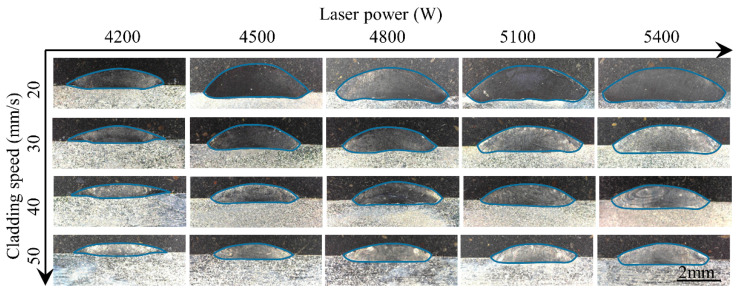
Cross-sectional surface of the clads under different laser powers and cladding speeds.

**Figure 7:**
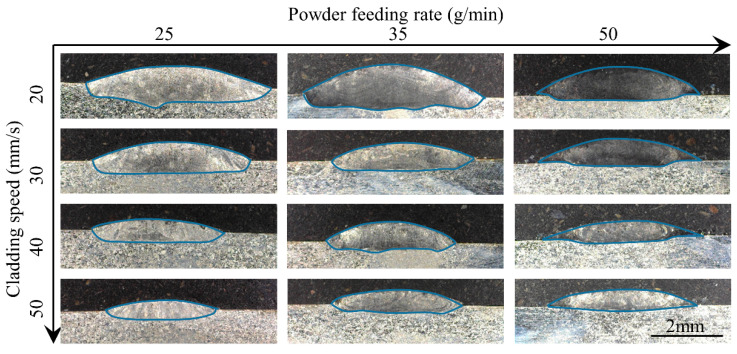
Cross-sectional surface of the clads under different powder feeding rates and cladding speeds.

**Figure 8:**
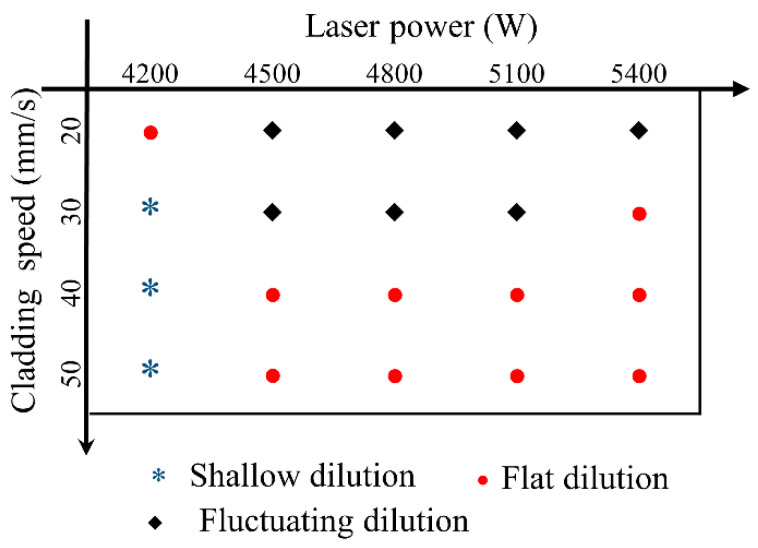
State diagram for dilution of processes under different cladding speeds and laser powers.

**Figure 9:**
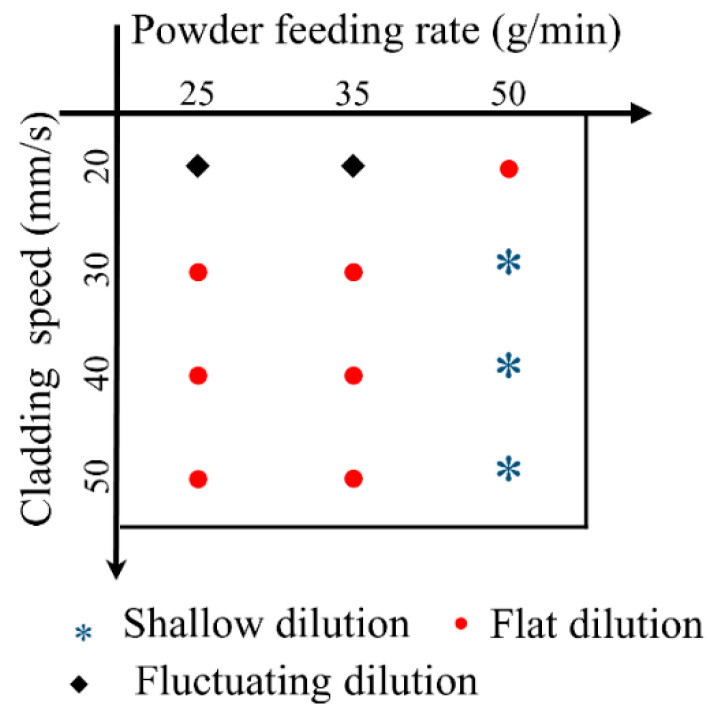
State diagram for dilution of processes under different cladding speeds and powder feeding rates.

Figure 6 and Figure 7 show the cross sections of the clad under different process parameters. Here, the nine geometrical characteristics explained in Section 2 are employed. The wetting angle is an important feature of the clad morphology and determines the lapping quality of multi-track and multi-layer cladding. If overlapping the clads are too convex, the likelihood of internal porosity is very high. In this study, the contact angle is less than 50°, indicating a good bonding strength between the adjacent clads in the multi-track cladding. The dilution ratio is a crucial parameter to evaluate the quality of the clad since it influences the chemical composition of the coating and the bonding strength between the clad and the substrate [29]. Distinguished by the morphology of the solid–liquid interface fusion line of the cross section, the melt pool can be divided into three types: shallow dilution (such as 50 mm/s and 50 g/min), flat dilution (such as 50 mm/s and 4800 W), and fluctuating dilution (such as 20 mm/s and 25 g/min). Figure 8 and Figure 9 show the three types of dilution under different processing parameters. It is obvious from Figure 8 and Figure 9 that the dilution of the flat bottom is the main form, which mainly occurs in the processes of high cladding speed, high laser power, and low powder feeding rate.

**Figure 10:**
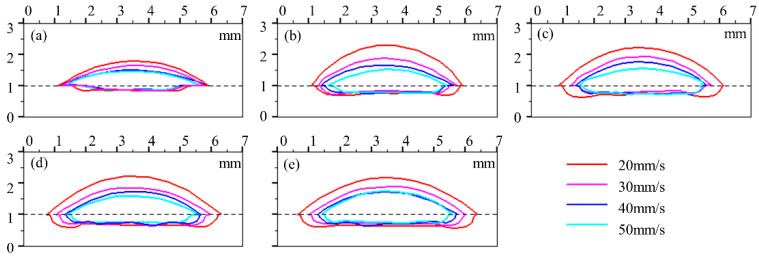
Profiles of the clad under different cladding speeds with laser powers of (**a**) 4200 W, (**b**) 4500 W, (**c**) 4800 W, (**d**) 5100 W, and (**e**) 5400 W.

**Figure 11:**
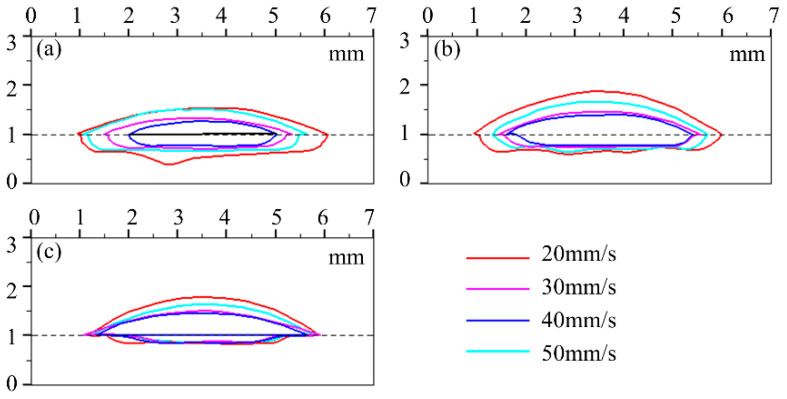
Profiles of the clad under different cladding speeds with powder feeding rates of (**a**) 25 g/min, (**b**) 35 g/min, and (**c**) 50 g/min.

In order to show the variation of the melt pool shape influenced by laser power and cladding speed, the profiles of the melt pool under the same laser power of four variations of cladding speed were plotted in a unified coordinate system within the same Figure, and the results are shown in Figure 10. Similarly, Figure 11 shows the morphologies of the clad under the same powder feeding rate of four variations of cladding speed. It can be seen from Figure 10 and Figure 11 that the effect of cladding speed on the clad morphology is mainly shown in clad height *h_c_* and clad width *W*, while there is a relatively small impact on penetration depth *h_m_*. The morphology of the clad is the result of the spatial and temporal evolution of the gas–liquid interface and the solid–liquid interface. The gas–liquid interface is the boundary of the melt pool that accumulates the input of powder materials and laser energy simultaneously. As the speed decreases, the accumulated laser energy for melting and the mass of the powder particles injected per unit of length on the interface reduce, resulting in a high sensitivity of *W* and *h_c_* to the cladding speed.

## 4. Discussion

### 4.1. Clad Areas of A_c_, A_m_, and A

**Figure 12:**
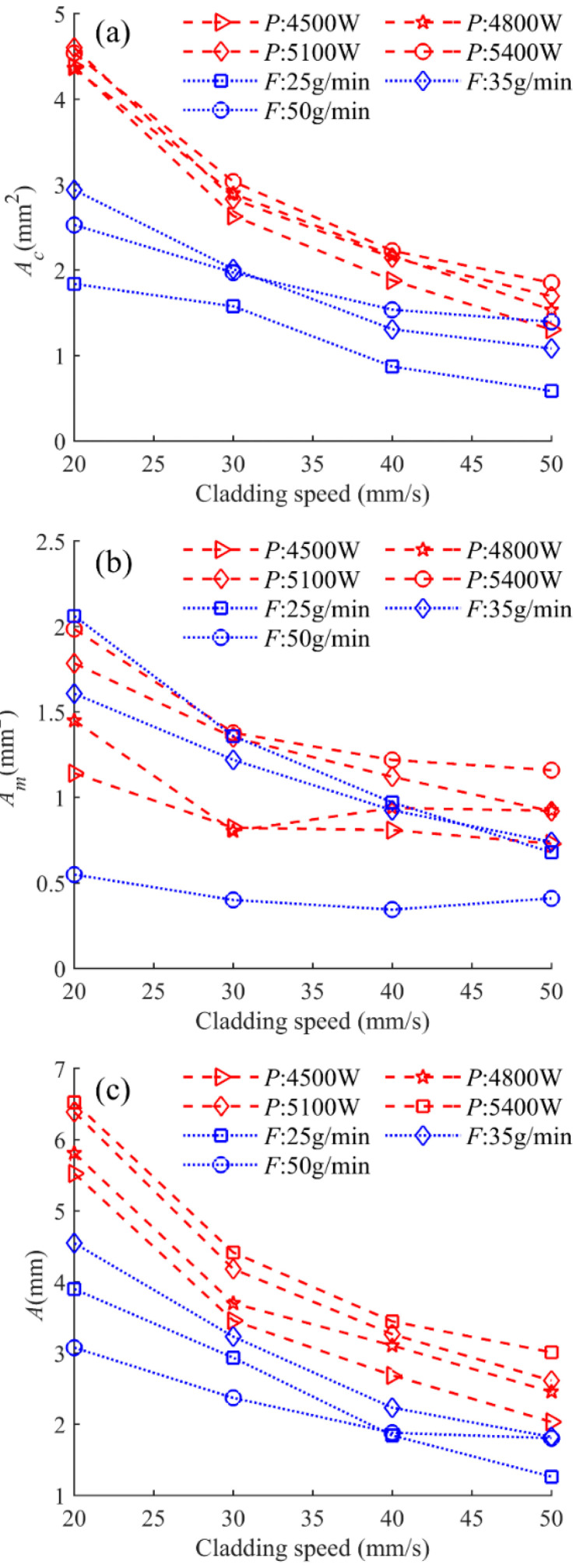
Clad areas of (**a**) *A_c_*, (**b**) *A_m_,* and (**c**) *A* as a function of cladding speed under different laser powers and powder feeding rates.

**Figure 13:**
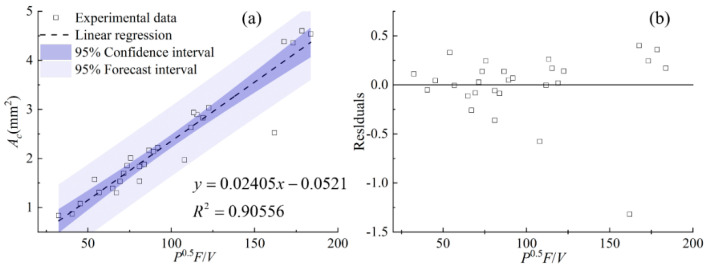
(**a**) *A_c_* plotted against combined parameter *P*^0.5^*F*/*V*, (**b**) the residuals.

**Figure 14:**
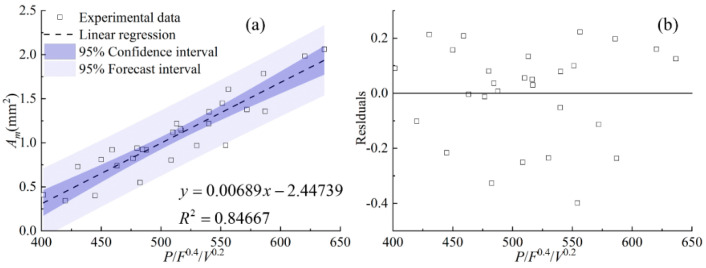
(**a**) *A_m_* plotted against combined parameter *P*/*F*^0.4^/*V*^0.2^, (**b**) the residuals.

**Figure 15:**
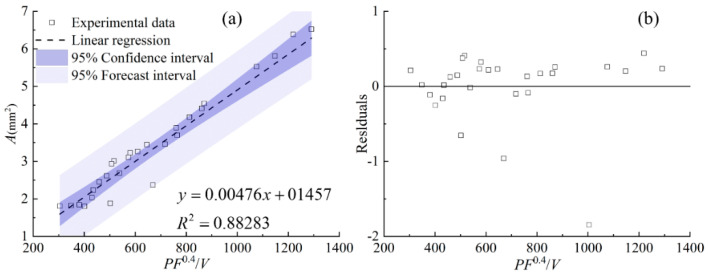
(**a**) *A* plotted against combined parameter *PF*^0.4^/*V*, (**b**) the residuals.

Figure 12 shows the relationship between the area of the clad above the substrate *A_c_*, the area of the molten substrate *A_m_*, the total area of the cross section *A,* and the cladding speed under different process parameters. It can be seen from Figure 12 that with the increase in scanning speed, the clad areas of *A_c_*, *A_m_*, and *A* generally show a decreasing trend, except for *A_m_* under the powder feeding rate of 50 g/min. When the powder feeding rate is relatively high, the effect of the cladding speed on *A_m_* is slight. The linear regression models between the combined parameters and the clad areas of *A_c_*, *A_m_*, and *A* are an effective way to quantify the process parameters and the clad morphology. Figure 13, Figure 14 and Figure 15 display the statistical reliance of the clad areas of *A_c_*, *A_m_*, and *A* along with the combined parameters. The large values of the linear regression coefficients and the residuals shown in Figure 13b, Figure 14b and Figure 15b testify of the correctness of the linear regression models.

As shown in Figure 13, the combined parameter *P*^0.5^*F*/*V* has a linear regression relationship with the area of the clad above the substrate *A_c_*, together with a relatively high regression coefficient of 0.91. This is consistent with previous work [14,17,21,25]. It is noteworthy that the areas of *A_m_* and *A* can be linearly regressed by the combined parameters of *P*/*F*^0.4^/*V*^0.2^ and *PF*^0.4^/*V* with relatively high regression coefficients of 0.85 and 0.88, showing a good correlation. The clad areas are crucial characteristics of the clad morphology, which can be used to evaluate the laser energy distribution between substrate and powder. In the process of coaxial transmission of powder and laser, there is only a part of the laser passing through the powder cloud and acting on the substrate to form a molten pool. Simultaneously, the powders are continuously captured by the molten pool, and then the laser directly acts on the surface of the captured powder. As a result, *A_c_* is larger than *A_m_*. Keeping the powder feeding rate constant, when the laser power increases, the energy reaching the substrate through the powder cloud increases, and thus the temperature of the powder heated during transmission and the temperature of the substrate increase, causing the increase in *A_c_* and *A_m_*, synchronously. When the laser power is constant, the energy reaching the substrate decreases due to the increase in the powder feeding rate, and the mutual shading between the powders also leads to a decrease in powder temperature. As a result, there is a decreasing trend in *A_m_*. Increasing the powder feeding rate leads to an increase in powder accumulated on the melt pool surface, so the powder feeding rate plays a positive role in increasing *A_c_*. Comparing the combined parameters of *P*^0.5^*F*/*V* and *P*/*F*^0.4^/*V*^0.2^, it can be concluded that the effect of the cladding speed on the clad is mainly manifested in *A_c_*. This is because the changing cladding speed directly affects the energy and mass input into the gas–liquid interface. The formation of the solid–liquid interface is due to complex physical processes, causing *A_m_* to be relatively less affected by the cladding speed.

### 4.2. Clad Heights of h_c_, h_m_, and H

**Figure 16:**
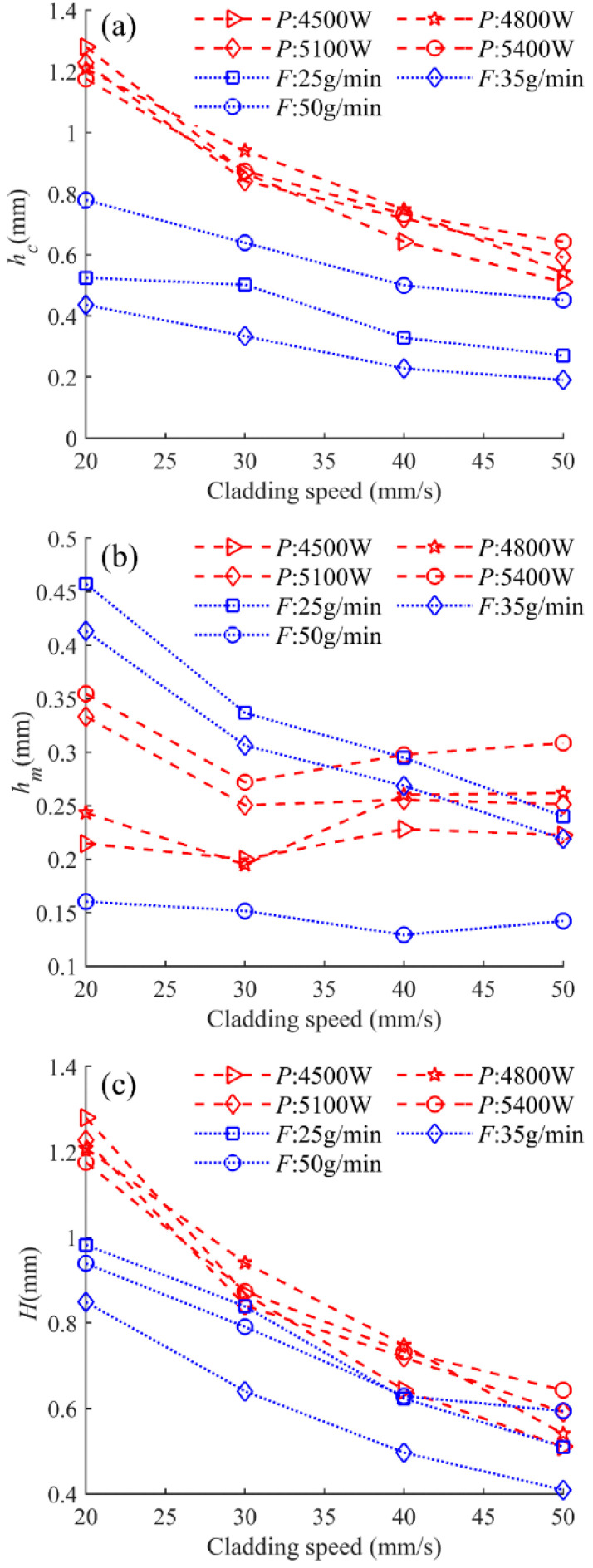
Cladding heights of (**a**) *h_c_*, (**b**) *h_m_*, and (**c**) *H* as a function of cladding speed under different laser powers and powder feeding rates.

Aiming at evaluating the effect of the main processing parameters on the height characteristics of the clad, three geometrical characteristics of the clad height above the substrate *h_c_*, the penetration depth *h_m_,* and the total height of the cross section *H* are employed. Figure 16 presents the dependence between *h_c_*, *h_m_,* and *H* and cladding speed under different laser powers and powder feeding rates. It is obvious that *h_c_* and *H* monotonically decrease with the increase in cladding speed. Different variation laws have been found between *h_m_* and cladding speed under different laser powers and powder feeding rates.

The heat and mass input into the surface of the molten pool, which is affected by many factors such as laser power, cladding speed, and powder feeding rate, leads to the complex evolution of the behavior of the gas–liquid interface. Increasing the powder feeding rate leads to more particle materials being added to the melt pool, and the input energy melts higher amounts of the added materials, resulting in the increased clad height. As a result, it is difficult to find a consistent pattern of variations of individual process parameters related to geometry morphology.

**Figure 17:**
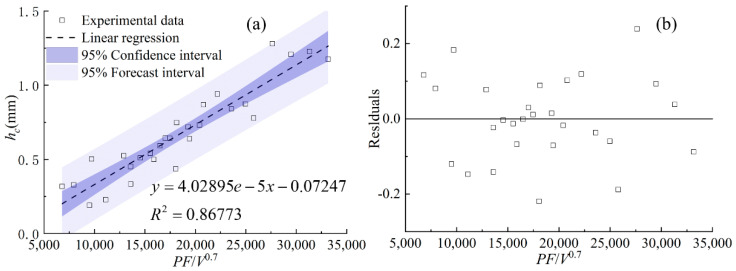
(**a**) *h_c_* plotted against combined parameter *PF*/*V*^0.7^, (**b**) the residuals.

**Figure 18:**
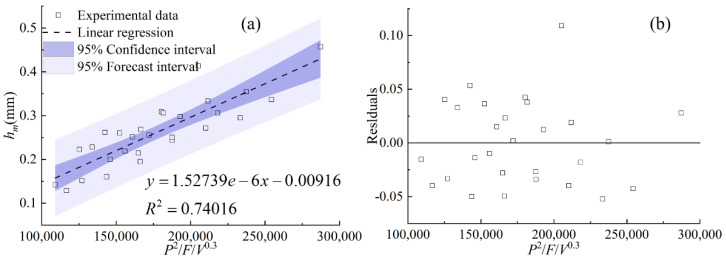
(**a**) *h_m_* plotted against combined parameter *P*^2^/*F*/*V*^0.3^, (**b**) the residuals.

**Figure 19:**
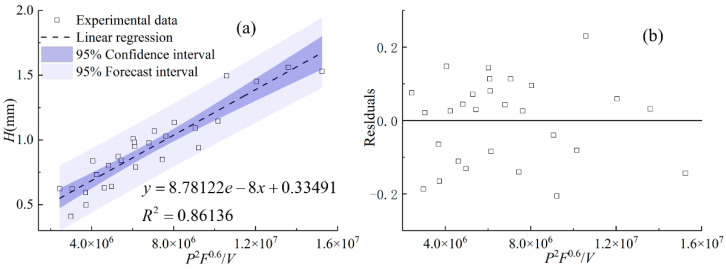
(**a**) *H* plotted against combined parameter *P*^2^*F*^0.6^/*V*, (**b**) the residuals.

Figure 17, Figure 18 and Figure 19 display the statistical reliance of the heights of *h_c_*, *h_m_,* and *H* along with the combined parameters, respectively. It is noticeable that the heights of *h_c_*, *h_m_,* and *H* are directly proportional to *PF*/*V*^0.7^, *P*^2^/*F*/*V*^0.3^, and *P*^2^*F*^0.6^/*V*, respectively, and the regression coefficients are 0.86, 0.74, and 0.86, respectively. The large values of the linear regression coefficients and the residuals plotted in Figure 17b, Figure 18b and Figure 19b testify of the correctness of the linear regression models. It is worth noting that laser power and powder feeding rate both have positive effects on *h_c_* and *H* while holding different weights. Both energy input and mass addition at the gas–liquid interface can increase the accumulation of powder on the melt pool surface, causing an increase in the heights of *h_c_* and *H*. The exponent related to the cladding speed of the combined parameters with the linear regression relationship of *h_m_* is 0.3, which is much smaller than that of *h_c_* and *H*, indicating the lower sensitivity of *h_m_* to the change of the cladding speed. This is due to the different formation mechanisms of the gas–liquid interface and the solid–liquid interface of the melt pool, which is in agreement with previous works [15,16].

### 4.3. Clad Width

**Figure 20:**
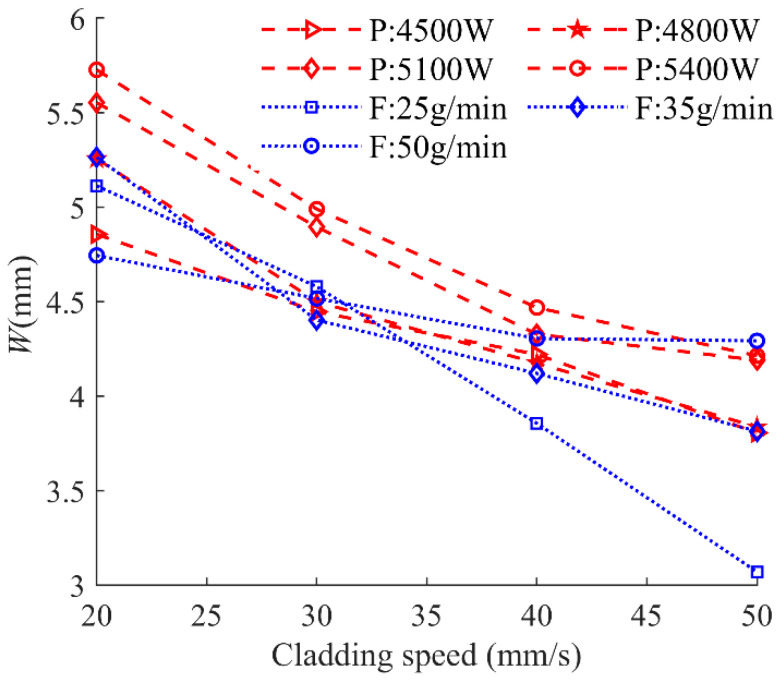
Cladding width as a function of cladding speed under different laser powers and powder feeding rates.

Figure 20 shows the behavior of clad width *W* as a function of cladding speed under different laser powers and powder feeding rates. It is obvious that there is a consistent decreasing trend for *W* along with the increase in cladding speed under different laser powers and powder feeding rates. Next, the linear regression model was established for quantitative analysis of the relationship between the processing parameters and *W*.

**Figure 21:**
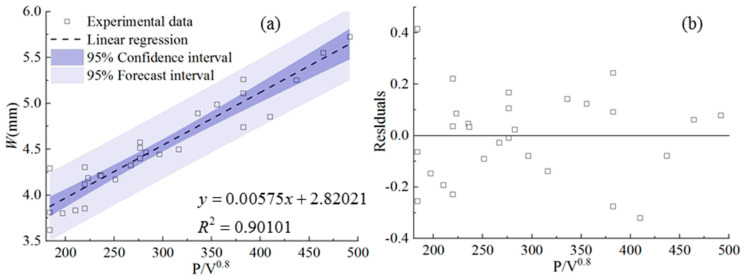
(**a**) *W* plotted against combined parameter *P*/*V*^0.8^, (**b**) the residuals.

The statistical dependence of *W* on the combined parameter is shown in Figure 21. It is noticeable that *W* is directly proportional to the combined parameter *P*/*V*^0.8^ with a linear regression coefficient of 0.90, which indicates the higher degree of the linear correlation ship between *W* and the combined parameter. It is noticeable that the parameter of the powder feeding rate is not involved in the expression of the combined parameter, indicating that the mass input on the gas–liquid interface has little effect on the formation of *W*. This conclusion is also confined by references [15,17,18], in which the combined parameters are written as *PV*^−1/2^, *P*^1/2^*V*^−1/5^, and *P*^3^*V*, respectively. It can be inferred that the width of the powder flow above the melt pool is larger than the width of the melt pool, which results in the powder flow doing little to the formation of the melt pool in the width direction.

**Figure 22:**
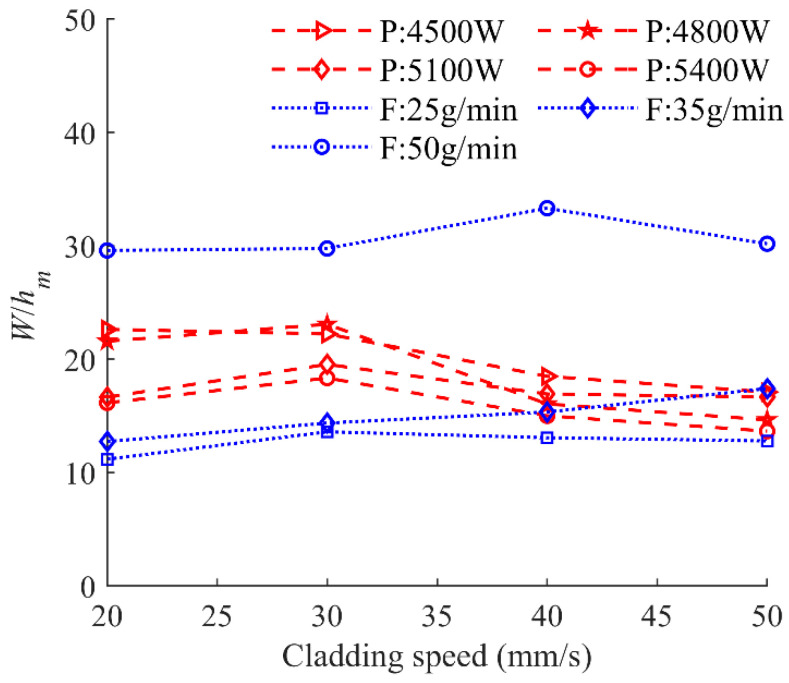
Ratio of *W* to *h_m_* as a function of cladding speed under different laser powers and powder feeding rates.

From previous studies [5,6,7], the Marangoni convection, which is affected by the material’s properties and the temperature distribution of the melt pool, is the fundamental mode of convection in the melt pool and can affect the shape of the melt pool dramatically. When the Marangoni convection in the melt pool is inward due to the positive temperature coefficient of the surface tension, the melt pool shape is characterized as narrow-deep. Otherwise, when the Marangoni convection in the melt pool is outward, the melt pool is wide-shallow [6]. Figure 22 displays the ratio of *W* and *h_m_* as a function of cladding speed under the laser powers and powder feeding rates. Within the selected parameters in the experiments, the ratio of *W* and *h_m_* is in the range of 11 and 34. This reflects that the effect of the outward Marangoni convection from the center to the edge plays a dramatical role in the formation of the melt pool. This is because of the negative surface tension coefficient of In718 [30] causing the outward direction of the thermos-capillary convection.

### 4.4. Dilution Ratio

**Figure 23:**
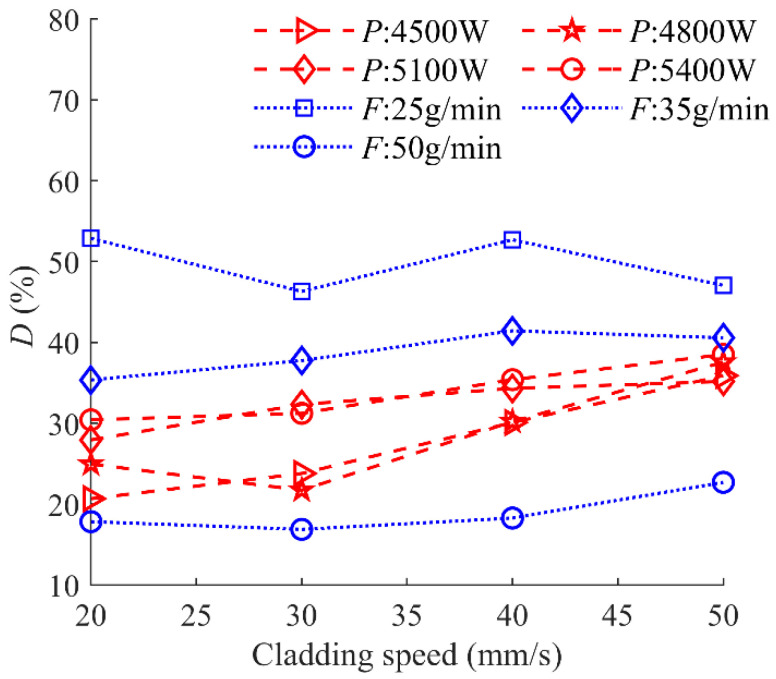
Dilution ratio as a function of cladding speed under different laser powers and powder feeding rates.

The dilution ratio *D*, acting as a crucial indicator for evaluating the quality of the cladding process, can be used to quantify the bond strength. Figure 23 shows the relationship between *D* and cladding speed under different laser powers and powder feeding rates. There is a slight increase in *D* with the increase in cladding speed. This can be explained by the previously established linear regression model in this study, which is related to the clad geometry of *h_m_* and *H*. The indices of cladding speed in the two regression models are 0.3 and 1, respectively, indicating that cladding speed has a “weak” effect on *h_m_*, while it has a ‘‘strong” effect on *H*. The geometrical characteristics of *h_m_* and *H* are the numerator and denominator of the *D* calculation formula, respectively, thus leading to the positive effect of cladding speed on *D*.

**Figure 24:**
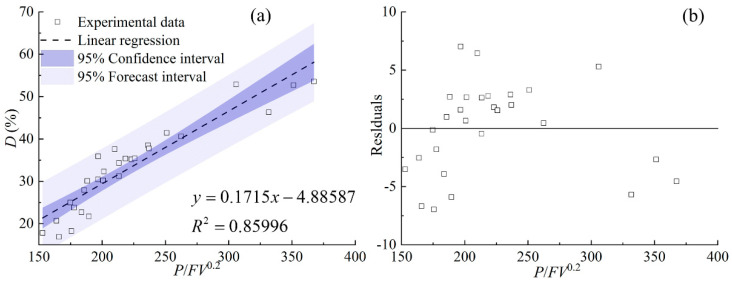
(**a**) *D* plotted against combined parameter *P*/*FV*^0.2^, (**b**) the residuals.

Figure 24 shows the characteristics of *D* as a function of the combined parameter. It is noticeable that *D* is directly proportional to the combined parameter *P*/*FV*^0.2^ with a linear regression coefficient of 0.90. The large linear regression coefficient indicates the high reliability of the statistical linear regression model, accompanied by the residuals plot (Figure 24b). The influence indexes of *α* and *β* in the linear regression model (*P*^α^*F*^β^*V*^γ^) are equal to 1, meaning the equally important effect of laser power and powder feeding rate when determining *D*. However, their effect on *D* is the opposite: with the increase in powder feeding rate, *D* increases, while *D* decreases with the increase in laser power. The influence of energy and mass input at the gas–liquid interface on *D* is a complex process. This is because the behaviors of laser energy input and powder mass addition affect *h_m_* and *H* simultaneously. Thus, the linear regression model provides a quantitative analysis method for predicting *D* under various processing conditions.

### 4.5. Wetting Angle

**Figure 25:**
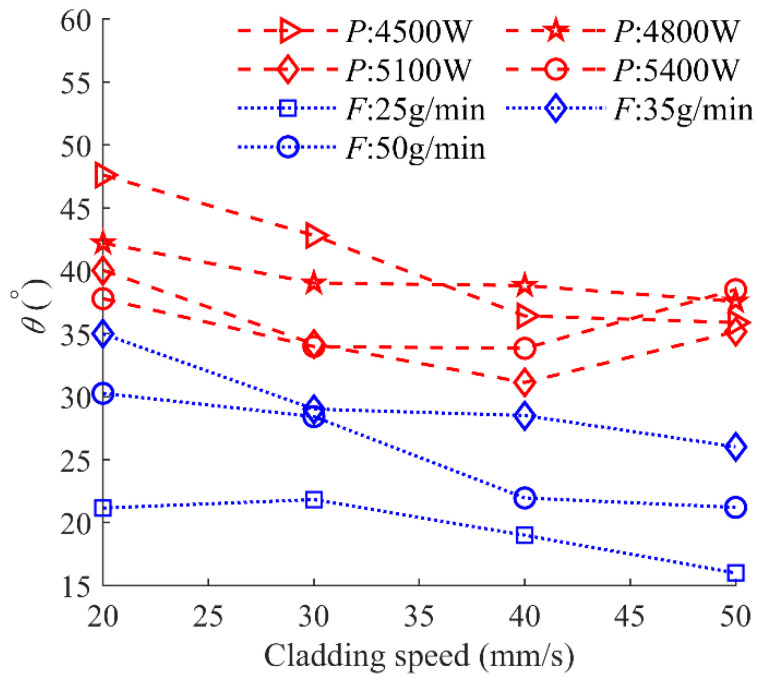
Wetting angle as a function of cladding speed under different laser powers and powder feeding rates.

The wetting angle *θ* is one of the most important characteristics of the clad, because it determines the overlapping morphology of multi-layer clads and the porosity of adjacent clads. According to reference [25], the wetting angle *θ* can be calculated by the equation written as *θ* = arctan (2*h_c_*/*W*). In this study, the wetting angles are less than 50°, indicating excellent bonding properties between adjacent clads. Figure 25 shows the behavior of *θ* as a function of cladding speed under different laser powers and powder feeding rates. There is a slight decrease in the wetting angle with the increase in cladding speed.

**Figure 26:**
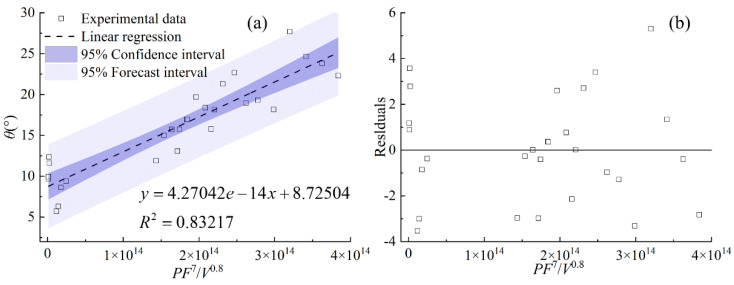
(**a**) *θ* plotted against combined parameter *PF*^7^/*V*^0.8^, (**b**) the residuals.

Figure 26 reveals the statistical correlation between the combined parameter *PF*^7^/*V*^0.8^ and the wetting angle. The correctness of the linear statistical model between the combined parameter *PF*^7^/*V*^0.8^ and *θ* is confirmed by a large correlation coefficient of 0.83, along with the distribution of the residuals (shown in Figure 26b). It is noticeable that the index of *F* is seven times that of *P*. As a result, it can be concluded that mass addition at the gas–liquid interface plays a major role in the formation of the wetting angle.

Without considering the physical process mechanism of the clad formation, the linear regression model provides a convenient way to establish the quantitative relationship between the complex multi-parameter process and the morphological characteristics of the clad. The linear regression models, which can be used to predict the geometry of the clad, show tremendous practical value for engineering applications.

## 5. Conclusions

In this paper, the effect of process parameters, including laser power, powder feeding rate, and laser cladding speed, within a high range on the geometric characteristics has been investigated. Quantitative and qualitative analysis of the relationship between the combined parameter *P*^α^*F*^β^*V*^γ^ and nine morphological characteristics of the clad has been carried out by the linear regression models, which are confirmed by large correlation coefficients and the analysis of residuals. The following conclusions can be drawn from the study:

1. The clad area can be used to evaluate the laser energy distribution between substrate and powder. The three parameters of the total area of the cross section *A*, the clad area above the substrate *A_c_*, and the area of the molten pool in the substrate *A_m_* are introduced to evaluate the evolution of the melt pool areas. The values *A_c_*, *A_m_,* and *A* can be linearly regressed by the combined parameters *P*^0.5^*F*/*V*, *P*/*F*^0.2^/*V*^0.4^, and *PF*^0.4^/*V*, which have been confirmed by large regression coefficients of 0.91, 0.85, and 0.88, respectively.
2. The clad height above the substrate *h_c_*, the penetration depth *h_m_,* and the total height of the cross-section *H*, which are dominated by the complex coupling of energy input and mass accumulation, are directly proportional to *PF*/^0.7^*V*, *P*^2^/*F*/*V*^0.3^, and *P*^2^*F*^0.6^/*V*, respectively, and the regression coefficients are 0.87, 0.74, and 0.86, respectively.
3. The ratio of clad width *W* and the penetration depth *h_m_* is in the range of 11 and 34, meaning that the Marangoni convection in the melt pool is outward and becomes the main driving force of the evolution of the width *W*. The cladding width *W* is mainly controlled by the laser energy input, confirmed by a linear relationship with the combined parameter *P*/*V*^0.8^.
4. The dilution ratio *D* and the wetting angle *θ* are the two most important characteristics of the clad, which can be used to evaluate porosity and bonding strength. The *D* and *θ* are directly proportional to the combined parameters *P*/*FV*^0.2^ and *PF*^7^/*V*^0.8^ with linear regression coefficients of 0.86 and 0.83, respectively.

## Figures and Tables

**Table 1:** Chemical Compositions (wt%) of Inconel 718.

Alloy	Ni	Nb	Mo	Cr	Al	Ti	Co	C	Si	S	Fe
IN718	52.98	5.07	3.12	19.1	0.5	0.95	0.049	0.039	0.093	0.003	Bal

## Data Availability

The authors confirm that the data supporting the findings of this study are available in the article. The raw data are available from the corresponding author upon reasonable request.
